# Analysis of the Possibility of Increasing the Load-Bearing Capacity and Fatigue Life of CFRP Material Mechanical Joints

**DOI:** 10.3390/ma18163735

**Published:** 2025-08-09

**Authors:** Angelika Arkuszyńska, Marek Rośkowicz

**Affiliations:** Faculty of Mechatronics, Armament and Aerospace, Military University of Technology, 00-908 Warszawa, Poland; marek.roskowicz@wat.edu.pl

**Keywords:** carbon composite, quasi-isotropic composite, mechanical fasteners, bearing stresses, load-bearing capacity, fatigue life

## Abstract

Achieving a high load-bearing capacity and fatigue life of joints of composite structures is possible with the use of mechanical fasteners. The aim of this research was to search for effective methods of increasing the load-bearing capacity of mechanical joints of CFRP components. A CFRP composite was made from carbon fabric (KORDCARBON, Czech Republic) using vacuum bag technology. Riveted and bolted joints were evaluated. The pressures exerted on the composite parts during assembly were measured. The values of pressures that cause permanent deformation of the tested composite were estimated. It was shown that the onset of failure of riveted joints is related to the pressures of the shanks of these fasteners on the holes. The load capacity of bolted joints also depends on the pressures of bolt heads on the composite elements. The value of bearing stresses in the pivot-loaded composite was determined. A tomographic study was conducted to determine the damage caused by their interaction. It was shown that the application of a rare-earth adhesive between the threaded bolt shank and the hole results in an increase in the load-bearing capacity of bolted joints by about 10%. A similar increase in strength is observed when the bolt assembly torque is increased.

## 1. Introduction

Carbon fibers and advanced polymer resins have become some of the most popular materials used in the manufacture of aircraft, both military and civilian, over the past few decades. In military airplanes and helicopters, composites can make up as much as one-third of the structural weight and include key components such as wings, control surfaces and the radar dome. In civilian structures, in composites in the past, secondary components were manufactured, but now primary components are also being produced [1,2]. CFRP composites enable 15–30% structural weight reduction, leading to 20–25% better fuel efficiency and lower emissions in aircraft like the Boeing 787 and Airbus A350 [3]. The rapid growth in the use of composite materials is due to their high stiffness, tensile strength, low density, chemical resistance, tolerance to elevated temperatures and low thermal expansion. These properties are made possible by the fact that by properly mixing the components of composite materials, the properties of the final product can be significantly improved [4,5,6]. Composite materials are more resistant to fatigue than any other structural materials, which can be induced in the case of aircraft by repetitive takeoff cycles and landings or aircraft operation in varying weather conditions.

Composite materials usually consist of layers arranged in a specific arrangement. Reinforcement fibers in individual laminas can be continuous or short, arranged in one or more directions, and randomly oriented in two or three dimensions [7]. Composites described as monotropic or otherwise unidirectional are those in which the stiffness and strength along the direction of the fibers (x_1_) are much higher than in directions transverse to them (x_2_ and x_3_) [8]. Properties that are more balanced and similar in the x_1_ and x_2_ directions are orthotropic composites. Fibers interlaced in two directions enhance strength and stability in multiple directions [9,10]. A quasi-isotropic composite, in the case of aerospace structures with complex stress states, is more resistant to operational damage. In addition, changing the orientation of successive layers of reinforcement by rotating them relative to each other allows constant properties regardless of changes in the reference system [11]. The disadvantage of both orthotropic and quasi-isotropic composites is the low stiffness and strength outside the layering (x_3_ direction) due to the flat arrangement of the fibers.

One of the most significant advances in the field of composite materials for the aerospace industry is the manufacture of complex parts in the form of a single component without joints, which can positively affect not only the strength of the structure but also reduce production costs [12]. Unfortunately, aerospace structures are such complex objects that at the current level of development of aerospace parts manufacturing technology, it is impossible to eliminate the joints of composite parts.

The three basic methods of joining fibrous composites to each other or to other structural materials are mechanical joints, adhesive bonding or a combination of these, referred to as hybrid joints. Adhesive bonding does not compromise the structure of the composite and does not add additional weight to the components being joined, but there are a number of disadvantages that exclude them from first-class aerospace applications because of the safety aspect and the difficulty of effectively diagnosing these joints in the process of aircraft operation [13]. In addition, adhesive joints are prone to aging and have limited resistance to high temperatures. Moisture softens the adhesive, impairs its mechanical properties and accelerates interphase damage. Increased temperature, on the other hand, changes the laws of cohesion and can cause hardening after curing or brittleness [14,15]. Weaknesses of adhesive joints are also related to the requirements of the technological process for their formulation, including requirements for surface preparation, technological pressures or thermal conditions for curing adhesive joints [16,17]. Hence, bonding is currently a rational method of joining primarily thin-walled components in so-called sandwich structures.

Hybrid (mechanical–adhesive) joints remain solutions that still require additional research in the area of effective load transfer between adhesive and mechanical joints and the effect of two-stage joint failure [18]. Hybrid joints can be subject to multiple interacting damage mechanisms, such as adhesive debonding, fiber breakage, fastener failure and net cross-section damage, which complicate prediction and control [19,20].

The third type of joining of composite parts is the use of mechanical fasteners. This type of joining, using rivets or bolts, can be particularly useful in repairing highly stressed composite airframe structures. Riveted and bolted joints are characterized primarily by the ability to carry high loads, technological susceptibility, resistance to moisture and temperature changes, the possibility of disassembling the elements and, incidentally, an easier certification process for this type of solution [21,22]. Challenges associated with the implementation of the assembly of composite elements with mechanical fasteners can be the quality of the assembly holes (made by drilling techniques) in the composite sandwich material and the guidelines associated with the placement of fasteners in seams or rows, which are different from those for metal elements [23].

At the production level, the bolt fasteners used in the assembly of composite structures of modern aircraft, among others, are special blind fastener systems of the Hi-lok or Jo-bolt type. Unfortunately, fasteners of this type are dedicated to joining a package of elements with a very strictly defined thickness, hence the need for a large assortment of these fasteners. In addition, these fasteners are expensive and difficult to access, and they are distributed primarily to certified centers responsible for maintaining aircraft airworthiness. It is also necessary to have specialized and expensive tooling to install them [24,25,26]. Considering the needs of the emergency repair system for aircraft, among other things, under conditions of time and logistical deficit, the possibility of using full-threaded nuts and bolts should also be considered.

The composite elements used in the assembly of mechanical fasteners are damaged in three main ways: first, as a result of tensile stresses, the composite material in the critical section is damaged; second, as a result of shearing stresses, the composite material is sheared towards the edge of the composite element; and third, the composite material subjected to bearing stresses at the material interface between the mechanical fastener shank and the walls of the assembly hole is damaged. The most desirable outcome in the process of machinery operation, including aircraft, due to the possibility of diagnosing damage, is the gradual destruction of the material under bearing stresses [26]. The destruction process includes several modes of micromechanical damage ranging from matrix cracking to the buckling of destabilized reinforcement fibers and further delamination of the composite material [27,28,29,30,31]. During this type of damage, crushing of the material and progressive ovalization of the hole can be observed with the unaided eye.

The purpose of this research was to search for solutions by means of which the load-bearing capacity and fatigue life of riveted and bolted composite material joints can be increased. Therefore, the influence of increasing the rivet swelling force and the bolt assembly torque was determined. The possibility of replacing special fasteners dedicated to composites with standard threaded bolts was considered after the application of a proprietary solution in the form of a low-viscosity adhesive material during assembly, limiting the destructive effect of thread tips on the side surface of the hole in the laminate. In addition, what the effect was of initial assembly pressures induced by mechanical joints on the load-bearing capacity and fatigue life of the joints was defined.

## 2. Materials and Methods

### 2.1. Joint Material

This study used a composite prepared on the basis of 160 g/m^2^ carbon fabric (KORDCARBON, Strážnice, Czech Republic) and aviation-certified epoxy resin L285 (MGS, Stuttgart, Germany) with a density of 1.19 g/cm^3^ mixed with a slow-crosslinking hardener 287 (MGS, Germany) at a ratio of 100:40. In order to obtain a composite with quasi-isotropic properties, 13 layers of fabric were laid according to the scheme [[0, 30, 60]_4_, 0]_T_. The composite was cured in two stages: in the first stage for 24 h at 20 °C using a vacuum bag technique and then at 80 °C for 6 h in a laboratory dryer (POL-EKO, Wodzisław Śląski, Poland). A composite material with a thickness of 2.5 ± 0.1 mm was obtained. For the purposes of this study, rectangular specimens were prepared with a width of 25 mm and a length of 100 mm.

**Figure 1:**
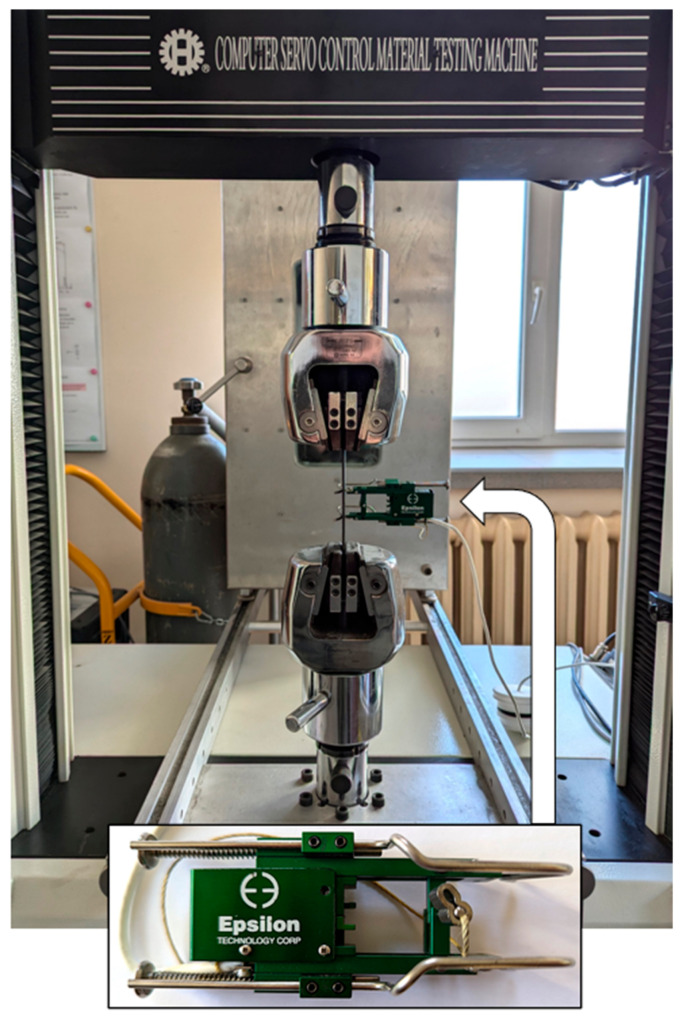
Composite specimen mounted in the jaws of the testing machine Hung Ta 2402 together with an Epsilon extensometer.

The Young’s modulus and tensile strength were determined using a 3542-025M-025-HT2 extensometer (Epsilon, Jackson, FL, USA) with a measuring base of 25 mm and a Hung Ta 2402 universal testing machine (Hung Ta, Taichung City, China), as shown in Figure 1. The Young’s modulus of the composite was equal to 38.75 GPa, while its tensile strength was 457 MPa.

### 2.2. Joint Preparation Process

**Figure 2:**
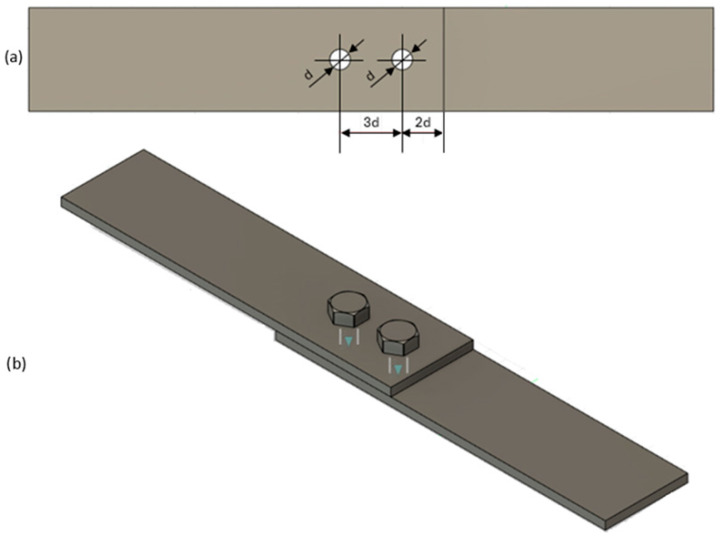
Mounting diagram of mechanical fasteners (d—diameter of the fastener shank): (**a**) diagram of the arrangement of the assembly holes; (**b**) view of the specimen used in the tests.

For testing the load-bearing capacity of the joints, single-lap riveted and bolted joints of composite components were prepared with AW2024T3 series aluminum alloy components with a thickness of 2 mm. A diagram of the assembly holes for the fasteners is presented in Figure 2.

**Figure 3:**
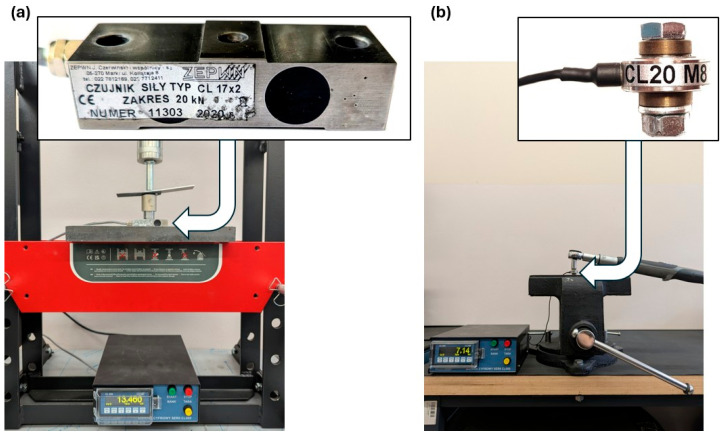
Measuring paths for (**a**) determining the swelling force in a riveted joint—CL17x2 sensor and (**b**) for determining the relationship between assembly torque and bolt preload force—CL20M8 sensor and BAHCO torque wrench.

Rivets with a diameter of 4 mm made of PA24 (AlCu2Mg) aluminum alloy and rivets with a diameter of 5 mm made of PA25 (AlCu4MnMg) aluminum alloy were used to make the joints [32]. Since a significant effect of the assembly process on the composite material was expected when rivets were used as fasteners, the swelling force was also measured. Force measurements were recorded using a CL17x2 force sensor (Figure 3a) with a measuring range of 20 kN (ZEPWN J. Czerwiński i Wspólnicy, Marki, Poland).

The bolt joints use bolts with the following diameters: M4, M5 and M6 class 8.8 and M6 class 12.9 bolts. The material properties of the bolts used are presented in Table 1. In the bolted joints, there are assembly pressures of the bolts and nuts on the material of the components to be joined; hence, the relationship between assembly torque and bolt preload force was determined using the CL20M8 force sensor (Figure 3b) with a measuring range of 10 kN (ZEPWN J. Czerwiński i Wspólnicy, Poland) and a torque wrench with a measuring range of 3–30 Nm (BAHCO, Enköping, Sweden).

**Figure 4:**
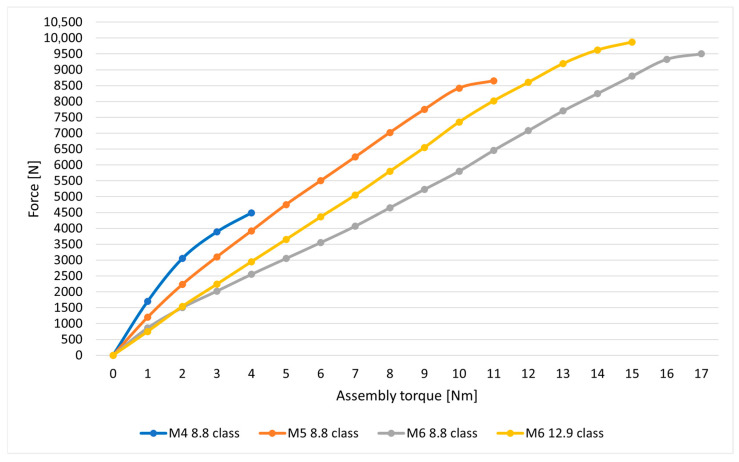
Relationship between bolt tension force and installation torque.

In the process of preparing the bolt joint, the nut was tightened by gradually increasing the torque until no further increase in force was observed, indicating plastic deformation of the bolt shank. The results of the measurements for the bolts used are presented in Figure 4. In the case of the M6 class 12.9 bolts, the yield strength of the bolt material was not determined due to limitations in the force sensor’s measurement range.

During the preparation of joints, assembly holes were made in the composite material, which induce the notch phenomenon and directly affect the strength parameters of the composite material. The effect of the hole’s impact on the mechanical properties of the CFRP composite was checked in a static tensile test of the composite specimen with a hole. In this test, specimens measuring 25 × 100 mm with a centrally drilled hole with a diameter of 6 mm were used. In addition, for the purpose of comparative analysis, the test checked the effect of installed bolts on the strength properties of the material. For this purpose, M6 bolts were placed in the assembly holes, tightening them with an assembly torque of 11 Nm, and the specimen thus prepared was subjected to static tension. In this way, we wished to define the effect of a pre-loaded mechanical fastener on the parameters of the CFRP material.

**Figure 5:**
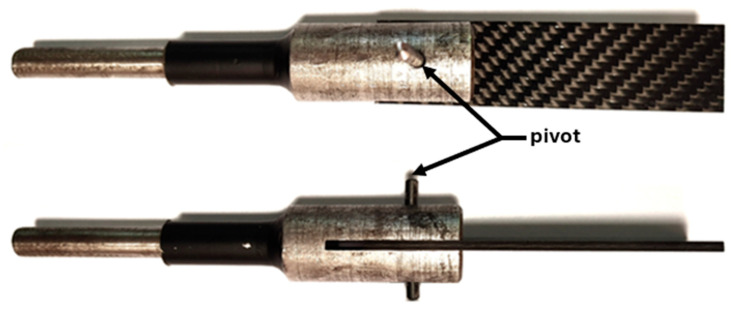
CFRP material specimen with hole and metal pivot prepared for tensile test.

The course of the destruction of the composite material due to the action of the mechanical fastener shank bearing stresses on the surface of the hole (without taking into account the installation pressures of bolts or rivets) was investigated using material specimens with a 4 mm diameter hole and a pivot placed in it, mounted in a special fork-shaped holder (Figure 5), which were gradually loaded to characteristic points on the tensile curve indicative of damage occurring in the composite material.

An analysis of the damage that occurred in the material because of the pressures generated by the pivot in the tensile test was conducted using a phoenix v|tome|x m metrology|edition X-ray microtomograph (ITA, Poznań, Poland).

**Figure 6:**
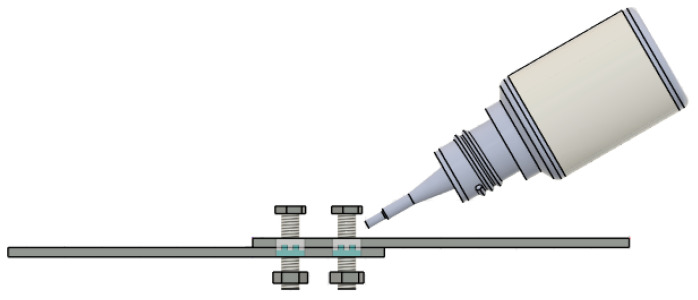
Method of assembling a bolt joint using adhesive plastic dispensed between the side walls of the assembly hole and the threaded bolt shank.

Since the fasteners used in this study were full-threaded bolts, the thread tips, when the joints were loaded, could initiate damage in the layered composite material. Therefore, in the search for effective ways to increase the load-bearing capacity of bolted joints, it was decided to test whether the application of a low-viscosity cyanoacrylate adhesive plastic between the threaded bolt shank and the lateral surface of the hole (Figure 6) during bolt assembly would have a beneficial effect on the load-bearing capacity of the joints. Tests in this regard were performed for lap joints for M4, M5 and M6 bolts.

Another action to increase the load-bearing capacity of bolted joints was to increase the assembly torque. Load-bearing capacity tests of the joints, in this regard, were performed using M5 bolts of class 8.8 and M6 bolts of class 12.9. In addition, fatigue life tests of the joints were also performed for this type of joint using an MTS 809 Axial machine (MTS, Eden Prairie, MN, USA). Durability tests were conducted in the range of alternating sinusoidal loads in the range of 3–10 kN per cycle with a load-change frequency of 5 Hz. The maximum load per cycle (10 kN) was equal to about 70% of the static load-bearing capacity of the joints.

## 3. Results

### 3.1. Influence of Notch on the Strength of CFRP Material

**Figure 7:**
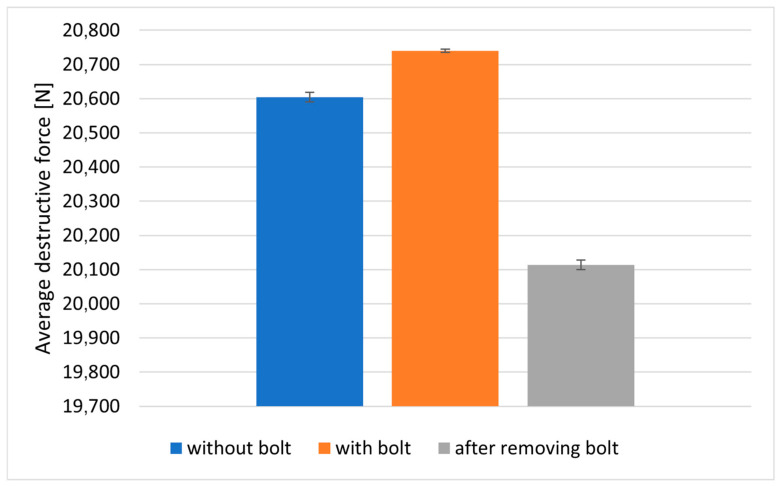
Comparison of the average values of forces causing damage to composite material in a static tensile test of specimens with 6 mm diameter holes.

The results of the tests on the failure stress values of the composite material are presented in Table 2 and Figure 7. The tests evaluated the effect of a notch in the form of an assembly hole without an installed bolt, with the installed bolt tightened to a controlled torque (11 Nm) and after removing the previously installed bolt.

To evaluate the change in the strength parameters of the tested material, after making assembly holes in it, the notch action factor was calculated. Comparing the results presented in Table 2 with the value of the failure stress for the material specimen without a hole (fn. 457 MPa), the notch action factor was equal to 1.054 for the hole without the fastener installed, 1.048 for the hole with the fastener installed and 1.079 after the fastener was removed.

**Figure 8:**
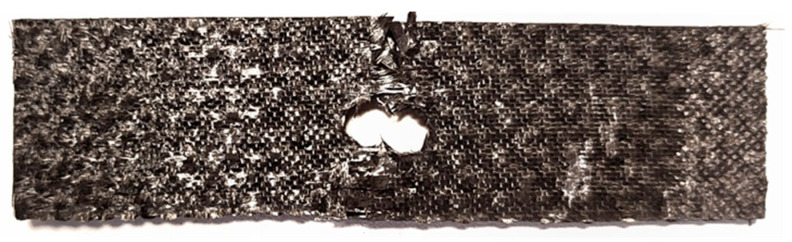
Damaged composite specimen with a 6 mm diameter hole.

The damage to the specimens with a hole was in the form of fiber rupture in the critical cross-section of the specimen (cross-section with a weakened hole—Figure 8). The assembly of bolts, their tightening with controlled torque and thus additional stress on the composite material in the vicinity of the assembly hole did not significantly affect the ad hoc strength of the composite material in the x_3_ direction.

### 3.2. Influence of Pivot Action on Composite Material

**Figure 9:**
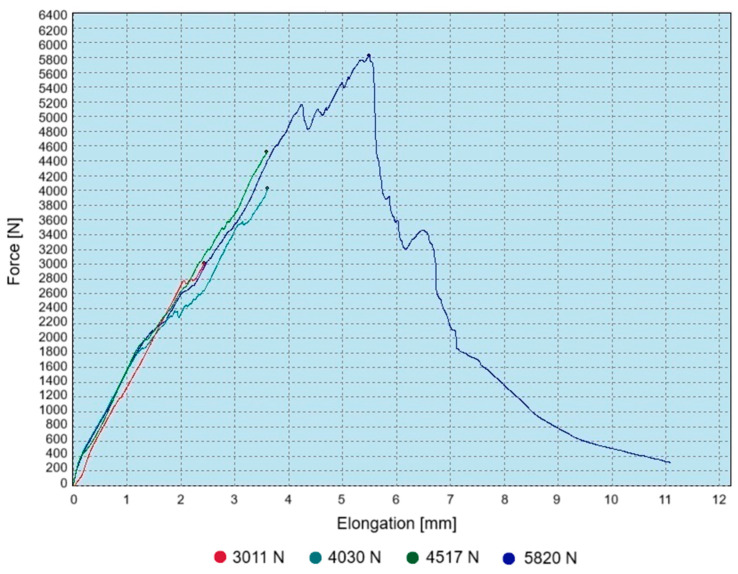
Tensile curves of composite specimens with a pivot placed in the hole along with maximum force values.

The test of resistance to pressure caused by the pivot on the wall of the assembly hole was performed on three specimens that were not completely destroyed. The load increase on the testing machine was stopped when the load value was reached, at which kinks were observed on the tensile curve that could indicate a change in the character of material failure. For the test specimens, it was a load equal to about 52%, 69% and 78% of the failure load, respectively. Using the fourth specimen, the value of the failure load was defined - load values presented in Figure 9.

**Figure 10:**
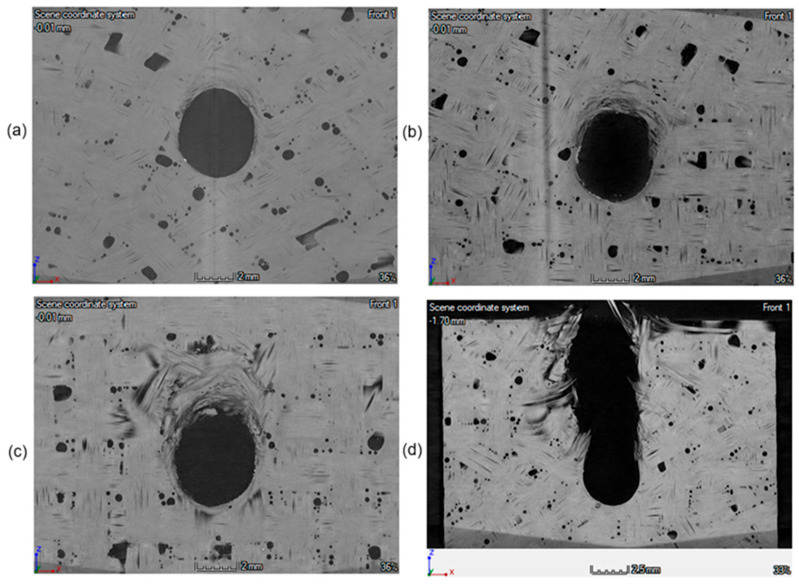
Tomograms of composite specimens loaded with force of (**a**) 3012 N, (**b**) 4030 N, (**c**) 4517 N, and (**d**) 5820 N.

The tomographic studies show that at bearing stresses of about 280 MPa the process of permanent deformation of the composite has already begun. In the subsequent tomographic stages, it was observed that as the load increases, the degree of degradation of the material increases, and also there is an increase in the ovalization of the assembly hole, as shown in Figure 10.

A correlation was found between the breakdowns in the characteristics shown in Figure 8 and the material condition observed during the tomographic inspection—Figure 10. The occurrence of such specific changes in the point graphs indicates successive stages and progression of bearing damage—progressive ovalization of the hole.

### 3.3. Load-Bearing Capacity of Riveted and Bolted Joints

**Figure 11:**
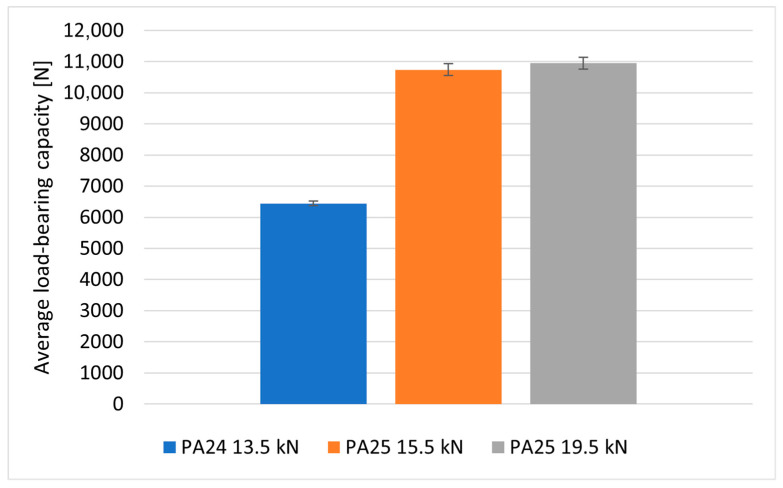
Comparison of the average load-bearing capacity of riveted joints.

The average value of the load-bearing capacity of the joints riveted for the five specimens using PA24 rivets with a diameter of 4 mm was equal to 6446 N. The failure of the joint consisted of shearing of the rivets, while no visible signs of damage to the composite material were observed on the joined parts (Figure 10a). Tests of riveted joints using PA25 rivets with a diameter of 5 mm were also performed for two types of specimens differing in the value of the swelling force of the rivets: in variant I, the value of the swelling force was equal to 15.5 kN, while in variant II, the value of the swelling force was equal to 19.5 kN. The load-bearing capacities of the joints for the specimens and the bearing stresses of the shanks on the lateral surfaces of the holes calculated from them are summarized in Table 3 and Figure 11. The assembly pressures exerted on the composite by the rivet heads during their swelling were determined using the recorded values of swelling forces.

**Figure 12:**
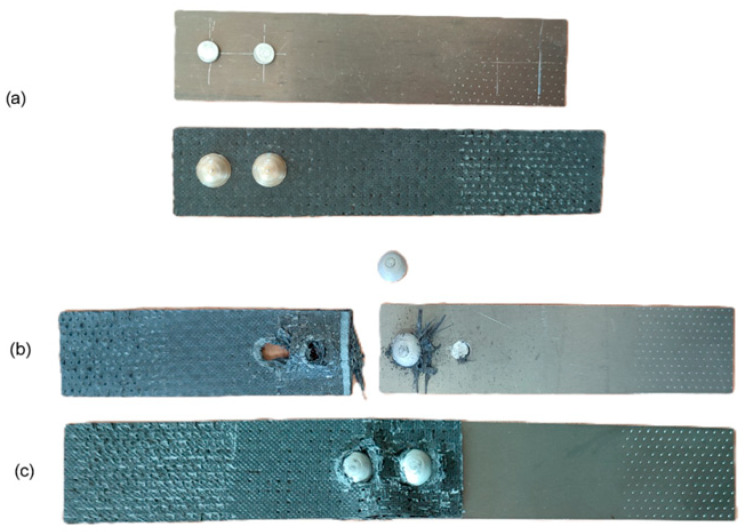
Failure form of rivet joints with rivets (**a**) with a diameter of 4 mm in PA24 aluminum alloy, (**b**) with a diameter of 5 mm in PA25 aluminum alloy swollen with a force of 15.5 kN and (**c**) with a diameter of 5 mm in PA25 aluminum alloy swollen with a force of 19.5 kN.

The application of different loads during the rivet swelling process did not significantly affect the load-bearing capacity of the joints but resulted in a different character of their failure; in joints with rivets swollen with a lower load, the rivets sheared (Figure 12b), while in joints with rivets assembled under higher loads, the rivets did not shear, but the joints failed as a result of the fasteners exceeding the allowable pressures on the lateral surfaces of the assembly holes (Figure 12c). This could have been caused by an increase in the strength of the rivet material due to the phenomenon of strain hardening.

With the knowledge of the values of assembly forces recorded during fastener assembly, the values of rivet head pressures on the composite in the direction of the x_3_ axis (x_3_—direction of layering in the composite) were determined.

The character of the destruction of the composite material for the joints with M4, M5 and M6 bolts was comparable. The damaged joints were disassembled, and the composite parts were visually inspected. To the unaided eye, it was easy to see that in the case of the M4 class 8.8 bolts and M6 class 12.9 bolts, that is, at pressures of about 150 MPa (designated pressure values in Table 4), the bolt heads led to permanent deformation of the composite material.

**Figure 13:**
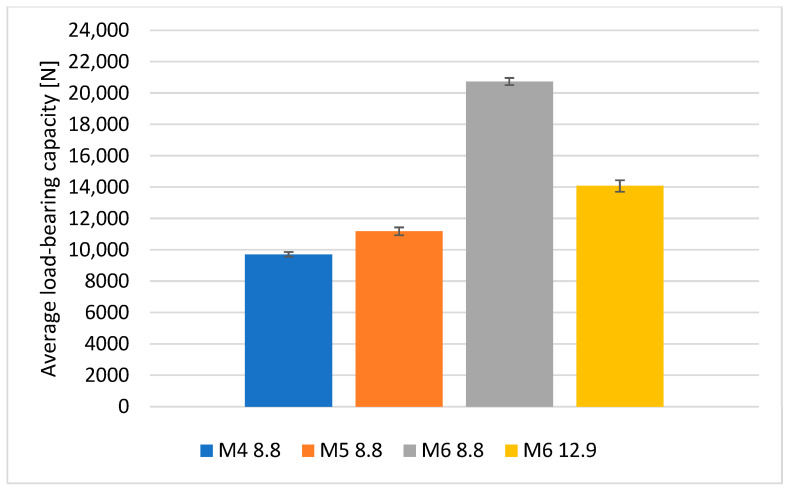
Comparison of the average load-bearing capacity of bolted joints.

A graphical representation of the differences in the load-bearing capacity of bolted joints depending on the fastener size and the assembly torque is shown in Figure 13.

### 3.4. Influence of the Use of Adhesive Plastic During the Assembly of Bolted Joints

**Figure 14:**
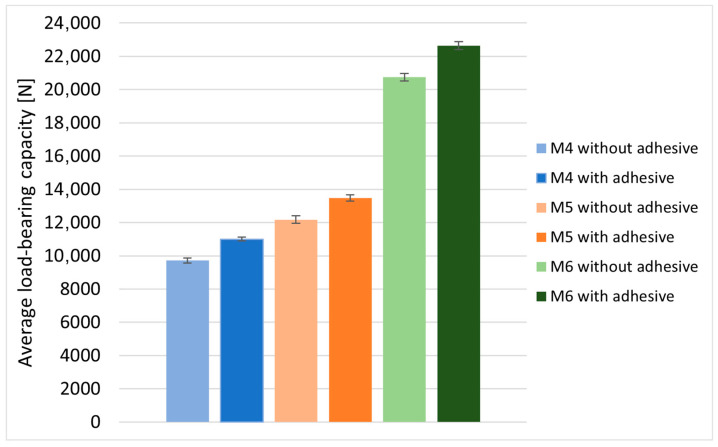
Comparison of the average load-bearing capacity of bolted joints assembled with/without adhesive material.

The effect of the use of a rare-earth adhesive plastic (assembly adhesive), applied between the threaded bolt shank and the side surface of the hole, on the load-bearing capacity of the joints during bolt assembly was assessed on bolted lap joints using M4, M5 and M6 class 8.8 bolts. With the same geometric and assembly parameters of the joint, the application of the adhesive plastic increased the load-bearing capacity of the joints. For joints with M4 bolts, the increase in the average load-bearing capacity of the joints was equal to 1285 N, which was 13%. For joints with M5 bolts it was 1200 N, which was about 10% compared to joints without adhesive plastic, while for M6 bolts the increase in the load-bearing capacity was 1900 N, which was 9%. The results of the tests performed are presented in Table 5 and Figure 14.

### 3.5. Influence of Assembly Torque on Load-Bearing Capacity of the Joints

For joints with M5 bolts, the effect of assembly torque on the load-bearing capacity of the joints was studied. The value of the torque with which the bolts were tightened was equal in the first variant to 5 Nm, while it was 8 Nm in the second. The results of the performed tests for joints with M5 bolts are presented in Table 6.

**Figure 15:**
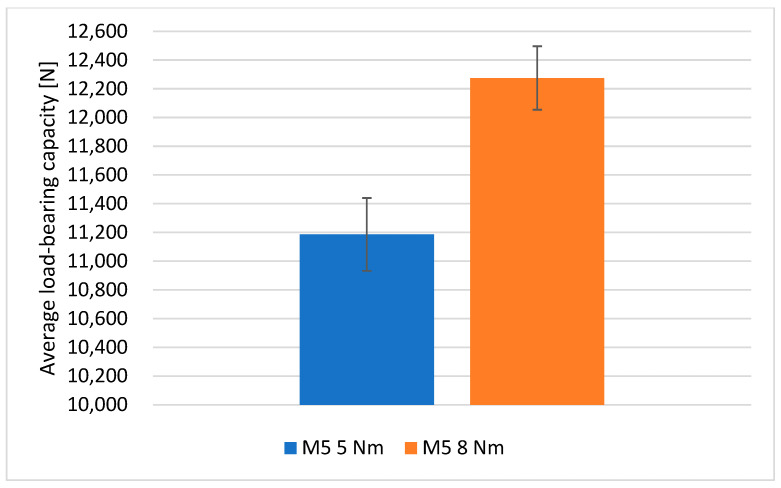
Comparison of the average load-bearing capacity of joints with M5 bolts tightened with a torque of 5 Nm and 8 Nm.

By increasing the assembly torque from a value of 5 to 8 Nm, an increase in the average load-bearing capacity of the joints of 9.73% was achieved, as shown in the graph in Figure 15.

**Figure 16:**
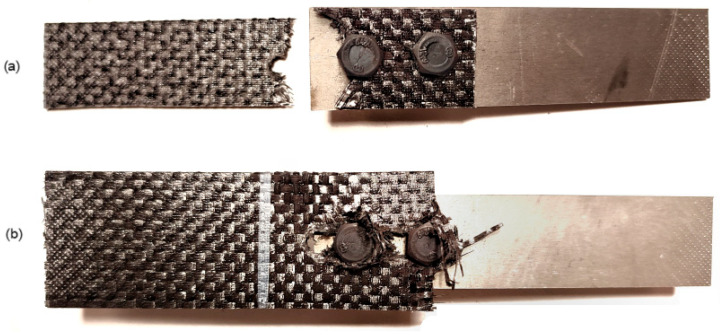
Damaged lap specimens with composite element widths of (**a**) 25 mm and (**b**) 35 mm bolted with M6 class 12.9 bolts.

The effect of changing the assembly torque of bolt fasteners on the load-bearing capacity of the joints was also evaluated for M6 class 12.9 bolts. The first tests were conducted for joints in which the fastened elements were 25 mm wide. In this case, increasing the value of the assembly torque from 5 to 15 Nm did not significantly affect the load-bearing capacity of the joints (Table 7), which was due to the manner of joint failure. The joints failed due to the decohesion of the material in the critical section of the elements to be joined (the section with the assembly hole). The value of the ratio of the width of the specimen to the diameter of the hole for this type of joint was equal to 4.1. Therefore, lap joints were prepared based on the same material and identical fasteners (M6 class 12.9 bolts) for elements with a width of 35 mm; then, the ratio of the width of the specimen to the diameter of the hole increased to a value of 5.74. Similarly to the joints with a width of 25 mm, the bolts in the joints with a width of 35 mm were also installed with two variants of torque. Damaged specimens with different composite element widths are shown in Figure 16.

The obtained load-bearing capacity values of the joints for the two types of joints (25 mm and 35 mm wide) are presented in Table 7.

**Figure 17:**
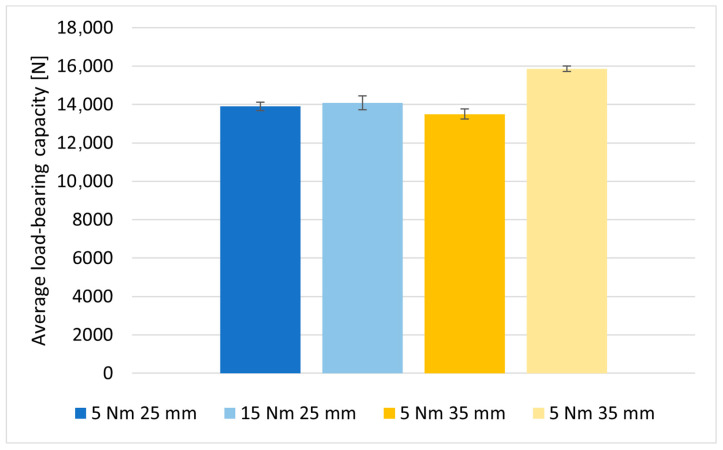
Comparison of the average load-bearing capacity of joints with M6 bolts of class 12.9 for joints with composite element widths of 25 mm and 35 mm.

For joints with a width of 35 mm, there was a very noticeable (Figure 17) increase in the average load-bearing capacity of the joints because of a higher value of torque during bolt assembly—by 2360 N, or almost 15%.

### 3.6. Influence of Assembly Torque on Fatigue Life of Joints

**Figure 18:**
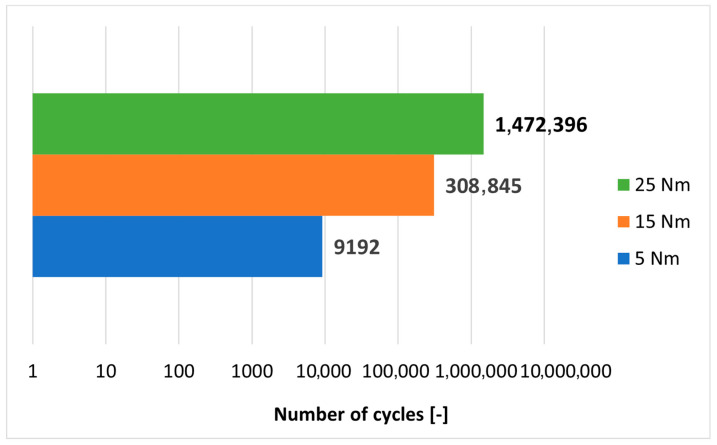
The relationship between bolt assembly torque and number of cycles in the fatigue test.

Since a clear relationship between the load-bearing capacity of the joints and the assembly torque applied to the bolt tightening was noted in the static tests, it was decided to supplement the tests in this regard with an evaluation of the fatigue life of the joints made under three variants of assembly torque. Lap joints with a width of 25 mm were prepared using bolt torques of 5 Nm, 15 Nm and 25 Nm. Durability tests were performed on an MTS 809 Axial testing machine (MTS, Eden Prairie, MN, USA) loading all joints with an asymmetrical sinusoidal alternating cycle at a frequency of 5 Hz in the load range of 3–10 kN per cycle. The maximum load per cycle was about 70% of the joint failure load. The fatigue life of the tested joints is presented in Figure 18 and Table 8.

**Figure 19:**
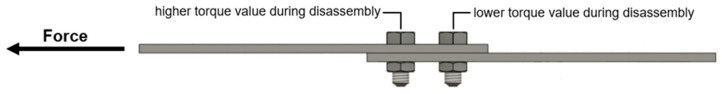
Difference in bolt disassembly torque values after fatigue testing.

Table 8 also shows the torque values recorded when the bolts were removed after the fatigue test was completed. It is shown that the applied alternating load caused loosening of the joints and a significant decrease in the bolt tightening torque values. It is observed that in a bolt node consisting of two fasteners, the loosening torque of one bolt was half that of the other, according to the following diagram in Figure 19.

## 4. Conclusions

The failure of riveted joints has been shown to be related to the bearing stresses of the shanks of these fasteners on the walls of the holes. The strength of bolted joints depends on the pressures of the heads on the composite elements. Pressures of these elements in the order of 150 MPa cause permanent deformation of the composites in the x_3_ direction without significantly weakening their strength.

The bearing stresses at which the first signs of permanent deformation of the composite occur were estimated to be about 280 MPa due to the action of the pivot on the lateral surfaces of the hole. The process of destruction of the joint is gradual, involving the cutting of the pin into the composite, which is accompanied by an increase in force caused by friction of the deformed composite against the walls of the handle, at the fork.

This study showed that it is more appropriate to use fasteners in the form of bolts rather than rivets for joining composite materials, as they allow higher load-bearing capacity of the joints.

An increase in the load-bearing capacity of bolted joints and their fatigue life was found for higher values of assembly torque during bolt tightening (higher head pressures). However, when increasing the value of applied torque, it is necessary to keep in mind the strength class of the bolts used—too high a torque value can lead to thread breakage.

The application of the rare-earth adhesive between the threaded bolt shank and the hole results in an increase in the load-bearing capacity of bolted joints of composite parts by about 10%.

## Figures and Tables

**Table 1:** Parameters of bolts used in the study.

Bolt Type		Maximum Force [N]	Cross- Sectional Area [mm^2^]	Yield Strength Determined Experimentally [MPa]	Literature Yield Strength [MPa]
M4	8.8	4490	7.75	579	640 ^1^
M5		8730	12.7	687	
M6		9600	17.9	536	
M6	12.9	not designated		not designated	1080 ^1^

^1^ [33].

**Table 2:** Values of forces/stresses causing failure of the composite material in static tensile tests of specimens with holes with a diameter of 6 mm.

Joint Parameters	Without Bolt	With Bolt	After Removing Bolt
1	21,275	20,800	20,800
2	21,358	21,000	19,200
3	18,783	20,500	19,058
4	21,000	20,000	21,392
5	16,200	21,400	16,542
Average destructive force	20,604 ± 1377	20,740 ± 463	20,113 ± 1308
Destructive stresses	433.77 ± 29	436.00 ± 10	423.43 ± 28
Notch action factor	1.054	1.048	1.079

Destructive force in Newtons [N], destructive stresses in Megapascals [MPa] and notch action factor—dimensionless quantity.

**Table 3:** Load-bearing capacity of riveted joints used in the tests and the value of pressures of rivet shanks and heads in the process of rivet assembly.

Joint Parameters	PA24 Ø 4 mm	PA25 Ø 5 mm	
	13.5 kN	15.5 kN	19.5 kN
1	6487	10,613	10,660
2	6363	10,893	11,483
3	6190	11,283	11,058
4	6577	10,200	10,330
5	6613	10,703	11,200
Average load-bearing capacity of joints	6446 ± 151	10,739 ± 371	10,946 ± 392
Bearing stresses of rivet shanks	322	430	438
Assembly pressures of rivet heads during their swelling	232	377	474
Form of joint damage	shearing of rivets	shearing of rivets and composite	shearing of rivets

Load-bearing capacity of joints in Newtons [N], bearing stresses in Megapascals [MPa] and assembly pressures in Megapascals [MPa].

**Table 4:** Load-bearing capacity of bolted joints.

Joint Parameters	M4	M5	M6	M6
	8.8			12.9
	4 Nm	5 Nm	11 Nm	15 Nm
1	9547	10,520	20,800	14,100
2	10,073	11,375	21,000	13,900
3	10,047	11,325	20,500	14,800
4	9600	10,727	20,000	14,400
5	9323	11,983	21,400	13,200
Average load-bearing capacity of joints	9718 ± 289	11,186 ± 509	20,740 ± 463	14,080 ± 722
Assembly pressures of bolt heads	147	124	63	157

Load-bearing capacity of joints in Newtons [N] and assembly pressures in Megapascals [MPa].

**Table 5:** Load-bearing capacity of bolted joints assembled with/without adhesive material.

Joint Parameters	M4		M5		M6	
	8.8					
	4 Nm		8 Nm		11 Nm	
	Without Adhesive Plastic	With Adhesive Plastic	Without Adhesive Plastic	With Adhesive Plastic	Without Adhesive Plastic	With Adhesive Plastic
1	9547	10,850	12,383	13,991	20,800	23,600
2	10,073	11,258	12,491	13,299	21,000	22,200
3	10,047	10,763	12,849	13,841	20,500	22,400
4	9600	10,980	11,492	13,225	20,000	22,600
5	9323	11,167	12,158	13,016	21,400	22,400
Average load-bearing capacity of joints	9718 ± 289	1100 ± 235	12,275 ± 442	13,475 ± 358	20,740 ± 463	22,640 ± 487
Assembly pressures of bolt heads	147		183		63	

Load-bearing capacity of joints in Newtons [N] and assembly pressures in Megapascals [MPa].

**Table 6:** Load-bearing capacity of joints with M5 bolts tightened with a torque of 5 Nm and 8 Nm.

Joint Parameters	5 Nm	8 Nm
1	10,520	12,383
2	11,375	12,491
3	11,325	12,849
4	10,727	11,492
5	11,983	12,158
Average load-bearing capacity	11,186 ± 509	12,275 ± 442
Assembly pressures of bolt heads	124	183

Load-bearing capacity of joints in Newtons [N] and assembly pressures in Megapascals [MPa].

**Table 7:** Load-bearing capacity of joints with M6 bolts of class 12.9 for joints with composite element widths of 25 mm and 35 mm (assembly torque of 5 and 15 Nm).

Assembly Torque	5 Nm	15 Nm	5 Nm	15 Nm
Width of the Composite Element	25 mm		35 mm	
1	13,600	14,100	13,700	15,600
2	14,300	13,900	13,800	15,700
3	14,000	14,800	13,200	16,100
4	14,100	14,400	12,900	15,900
5	13,500	13,200	13,900	16,000
Average load-bearing capacity of joints	13,900 ± 421	14,080 ± 722	13,500 ± 534	15,860 ± 277
Assembly pressures of bolt heads	57	157	57	157

Load-bearing capacity of joints in Newtons [N] and assembly pressures in Megapascals [MPa].

**Table 8:** Fatigue life of bolted joints tightened with different values of assembly torque.

Assembly Torque	Number of Cycles	Disassembly Torque
5 Nm	9192	0.00 Nm
		3.51 Nm
15 Nm	308,845	3.34 Nm
		6.05 Nm
25 Nm	1,472,396	7.60 Nm
		14.50 Nm

Torque in Newton-meters (Nm); number of cycles—non-dimensional quantity.

## Data Availability

The original contributions presented in this study are included in the article. Further inquiries can be directed to the corresponding author.

## Abbreviations

The following abbreviations are used in this manuscript:

CFRP	Carbon Fiber Reinforced Polymer
